# Travel-associated and autochthonous Zika virus infection in mainland France, 1 January to 15 July 2016 

**DOI:** 10.2807/1560-7917.ES.2016.21.32.30315

**Published:** 2016-08-11

**Authors:** A Septfons, I Leparc-Goffart, E Couturier, F Franke, J Deniau, A Balestier, A Guinard, G Heuzé, A H Liebert, A Mailles, JR Ndong, I Poujol, S Raguet, C Rousseau, A Saidouni-Oulebsir, C Six, M Subiros, V Servas, E Terrien, H Tillaut, D Viriot, M Watrin, K Wyndels, the Zika Surveillance Working Group in French departments and collectivities of the Americas, H Noel, MC Paty, H De Valk

**Affiliations:** 1 Santé publique France, French national public health agency, Saint-Maurice, France; 2 European Programme for Intervention Epidemiology Training (EPIET), European Centre for Disease Prevention and Control, (ECDC), Stockholm, Sweden; 3 Institut de Recherche Biomédicale des Armées, National Reference Laboratory for arboviruses, Marseille, France; 4 Santé publique France, French national public health agency, Regional unit (Cire) Provence Alpes Côtes d’Azur, Saint-Maurice, France; 5 Santé publique France, French national public health agency, Regional unit (Cire) Languedoc Roussillon Midi Pyrénées, Saint-Maurice, France; 6 Santé publique France, French national public health agency, Regional unit (Cire) Pays de la Loire, Saint-Maurice, France; 7 Santé publique France, French national public health agency, Regional unit (Cire) Centre Val de Loire, Saint-Maurice, France; 8 Santé publique France, French national public health agency, Regional unit (Cire) Auvergne Rhône Alpes, Saint-Maurice, France; 9 Santé publique France, French national public health agency, Regional unit (Cire) Alsace Lorraine Champagne Ardenne, Saint-Maurice, France; 10 Santé publique France, French national public health agency, Regional unit (Cire) Ile de France, Saint-Maurice, France; 11 Santé publique France, French national public health agency, Regional unit (Cire) Aquitaine Limousin Poitou Charentes, Saint-Maurice, France; 12 Santé publique France, French national public health agency, Regional unit (Cire) Bourgogne Franche Comté, Saint-Maurice, France; 13 Santé publique France, French national public health agency, Regional unit (Cire) Bretagne, Saint-Maurice, France; 14 Santé publique France, French national public health agency, Regional unit (Cire) Normandie, Saint-Maurice, France; 15 Santé publique France, French national public health agency, Regional unit (Cire) Nord Pas de Calais, Saint-Maurice, France; 16 The members of the group are listed at the end of the article

**Keywords:** Zika virus, surveillance, France

## Abstract

During summer 2016, all the conditions for local mosquito-borne transmission of Zika virus (ZIKV) are met in mainland France: a competent vector, *Aedes albopictus*, a large number of travellers returning from ZIKV-affected areas, and an immunologically naive population. From 1 January to 15 July 2016, 625 persons with evidence of recent ZIKV infection were reported in mainland France. We describe the surveillance system in place and control measures implemented to reduce the risk of infection.

From 1 January to 15 July 2016, 625 persons with evidence of recent Zika virus (ZIKV) infection were reported in mainland France. This large influx of ZIKV-infected travellers reflects the current epidemic of ZIKV infection in the French departments and collectivities of the Americas – Martinique, Guadeloupe, Saint Martin, Saint Barthélemy and French Guiana [1] – and coincides with the activity period (May to November) of the vector *Aedes albopictus* in mainland France. Because of an increase in the number of travellers from the French departments and collectivities of the Americas during the summer holidays, the risk of introduction and transmission of ZIKV in mainland France is at its height in the summer months of 2016. We describe the surveillance system and control measures implemented in mainland France to reduce this risk, as well as some preliminary results.

## Surveillance of Zika virus infection in mainland France

Surveillance of ZIKV infections has been integrated into the system implemented for chikungunya and dengue in mainland France, which has been in place since 2006 [2]. The objectives of the surveillance are to detect imported or autochthonous cases early and to prevent local transmission by the early implementation of vector control measures. An additional specific objective for ZIKV surveillance is to identify ZIKV-infected pregnant women, in order to ensure enhanced follow-up of their pregnancies in specialised centres, and describe their pregnancy outcomes.

The surveillance system comprises several components related to ZIKV infection:

• nationwide year-round notification of probable and confirmed cases of ZIKV infection (in place since 1 January 2016, mandatory since 5 June 2016);

• seasonal enhanced surveillance in administrative departments where the vector is established. From 1 May to 30 November, when the vector is active, all suspected imported cases must be immediately reported to the regional health authorities. Without waiting for laboratory confirmation, an entomological investigation is immediately carried out around the places visited by the patient during their likely viraemic period (defined as two days before until seven days after the onset of symptoms). According to the findings, appropriate vector control measures, comprising the elimination of larval breeding sites and spraying of larvicides (*Bacillus thuringiensis israelensis*) and adulticides (pyrethroids) [2,3], are implemented in an area of 200 m around these places;

• daily reporting from a network of laboratories of the results of Zika serological or RT-PCR tests to the French national public health agency. This allows catching up on confirmed cases which have not been reported through the notification system and the seasonal enhanced surveillance;

• notification of pregnancy outcomes for pregnant women infected by Zika virus, or possibly exposed to the virus through sexual or mosquito-borne transmission.

A suspected case of ZIKV infection is defined as a person presenting with rash, with or without fever and at least two of the following: arthralgia, myalgia or conjunctivitis/conjunctival hyperaemia, not explained by another medical condition. 

A probable case is a suspected case with anti-ZIKV IgM antibodies in serum sample(s). 

Cases are confirmed by serology (anti-ZIKV IgG antibodies confirmed by plaque-reduction neutralisation test, or fourfold increase in IgG titre or seroconversion) or by detection of viral nucleic acids in body fluids (blood, cerebrospinal fluid, urine, semen, saliva, etc.) by reverse transcription (RT)-PCR.

To characterise ZIKV infection, information on patients’ demographics, recent travel history and exposure, clinical presentation and symptoms are collected for each confirmed case.

Since January 2016, the National Reference Centre for Arboviruses in Marseille has contributed to diagnostic capacities for ZIKV in hospital and private medical laboratories by making available reference material, operating procedures and testing/diagnosis algorithms. The Ministry of Health has ensured the reimbursement of serology and RT-PCR tests for ZIKV, under certain conditions, through the National Health Insurance Scheme.

## Cases of Zika virus infection in mainland France

**Figure 1:**
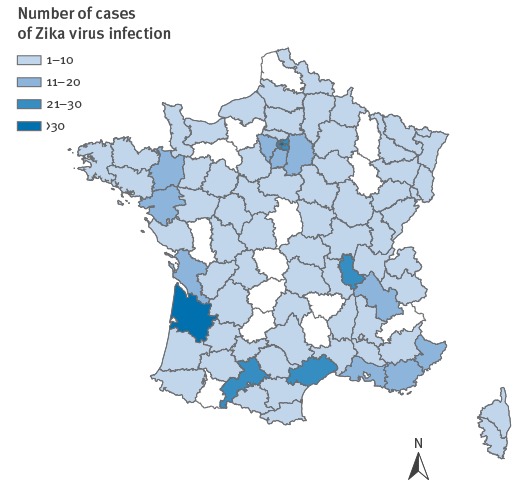
Cases of Zika virus infection by administrative department, mainland France, 1 January–15 July 2016 (n = 625)

Source: Santé publique France, French national public health agency, France, 2016.

From 1 January 2016 to 15 July 2016, 625 cases of ZIKV infection, 537 confirmed (86%) and 88 probable (14%), were reported (Figure 1).

Among the 625 cases, 617 (99%) reported recent travel to an area with active ZIKV transmission and 8 (1%) were infected after sexual intercourse with an infected traveller [4-6].

A total of 357 cases (57%) were female. The median age of the cases was 45 years (range: 2–84) (Table).

**Table:** Characteristics of cases of Zika virus infection, mainland France, 1 January–15 July 2016 (n = 625)

Characteristic	Number (%)
Sex	
Female	357 (57)
Age group in years	
< 10	6 (1)
10–19	15 (2)
20–29	83 (13)
30–39	155 (25)
40–49	106 (17)
50–59	122 (20)
60–69	109 (17)
≥ 70	29 (5)
Regions visited during the incubation period^a^	
French departments and collectivities of the Americas	527 (84)
Caribbean islands	28 (4)
South America	25 (4)
Central America	8 (1)
Asia	1 (0.2)
Pacific	1 (0.2)
Africa	1 (0.2)
Not documented	26 (4)
No travel	8 (1.3)
Complications	
Guillain–Barré syndrome	2 (0.3)
Meningoencephalitis	1 (0.2)
Hospitalisation	29 (5)
Viraemic cases^b^	156 (25)
Month of notification	
January	8 (1)
February	76 (12)
March	74 (12)
April	121 (19)
May	144 (23)
June	158 (25)
July^c^	44 (7)

^a^ During the two weeks before symptom onset.

^b^ In an area in which the vector *Aedes albopictus* is established and active.

^c^ Until 15 July 2016.

ZIKV infection was confirmed by detection of viral nucleic acids by RT-PCR in blood or urine for 487 (78%) cases, RT-PCR in blood or urine and serum IgM positivity for 36 cases (6%), seroconversion for two (0.3%) cases, detection of ZIKV RNA by RT-PCR in semen for 6 cases (1%) and in cerebrospinal fluid for 1 case (0.2%) with meningoencephalitis, by detection of neutralising antibodies against ZIKV for 5 cases (0.8%). For 88 (14%) cases, only a positive serological test (IgM) was available.

Clinical illness was reported in 570 cases (91%), 46 (7%) are still under investigation to obtain clinical information and 7 (1%) were asymptomatic.

Among the seven asymptomatic cases, three were tested because of a planned medically assisted procreation intervention (one woman, two men). One woman was tested because she had been in a ZIKV-epidemic area and wanted to get pregnant, one woman was tested during the investigation of an instance of likely sexual transmission of the virus and two women were tested because they had been exposed in an epidemic area and were pregnant. All asymptomatic cases were confirmed by detection of viral nucleic acids by RT-PCR (four in urine and three in blood).

Among the 570 cases with clinical illness, the most commonly reported signs or symptoms were rash (84%, n = 480), fever (64%, n = 367), arthralgia (64%, n = 367), myalgia (57%, n = 325) and headache (52%, n = 295). Only 20% (n = 112) reported conjunctivitis. Three cases had neurological complications: two had Guillain–Barré syndrome, one had meningoencephalitis [7].

Nine patients reported other neurosensitive symptoms including paraesthesia of the hands, arms or around the mouth (n = 4), hypoesthesia of the hands (n = 3), cutaneous hyperesthaesia (2/9).

Hospitalisation was required for 29 (5%) patients and there were no deaths. There were 16 pregnant women among the cases.

A majority (85%, n = 527) of confirmed imported cases of ZIKV infection were travellers returning from the French departments and collectivities of the Americas (327 from Martinique, 160 from Guadeloupe, 21 from French Guiana, 16 from Saint Martin and 3 from unspecified locations in the French departments and collectivities of the Americas). The remaining cases had returned from other Caribbean islands and Central or South American countries (Table).

**Figure 2:**
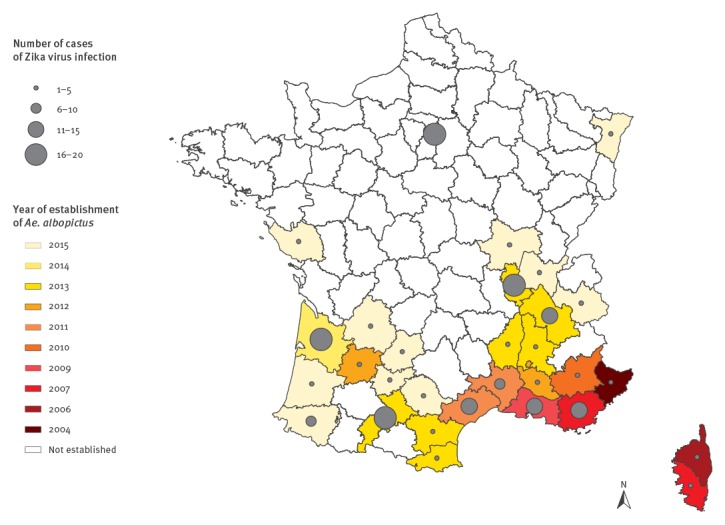
Establishment of *Aedes albopictus* in mainland France, by administrative department and year (2004–15), and number of cases of Zika virus infection since the start of the vector activity season, 1 May–15 July 2016 (n = 185)

Source: Santé publique France, French national public health agency, France, 2016.

On their return to mainland France, 185 (30%) had stayed in an *Ae. albopictus*-established area during the vector activity period (Figure 2), 84% (n = 156) of them were viraemic. The median delay between the onset of symptoms and date of return in an area with active vectors was two days (range: −7 to 10) with 82% (n = 128) of cases staying in those areas during the entire period of viraemia. Entomological investigations led to the implementation of vector control measures for 21% (32/156) of the cases. The median delay between onset of symptoms and implementation of vector control measures was 13 days (range: 4–58) and between notification and intervention 5 days (range: 2–38).

**Figure 3:**
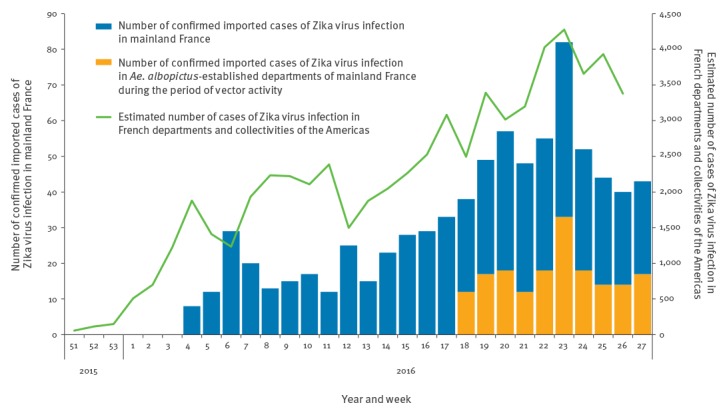
Imported cases of Zika virus infection in mainland France (weeks 4–27 2016^a^, n = 617), imported cases staying in an *Aedes albopictus*-established area in mainland France during the period of vector activity (weeks 18–27 2016^b^, n = 183) and estimated number of cases in the French departments and collectivities of the Americas (week 51 2015–week 26 2016^c^, n = 62,825)^d^

^a^ 25 January–10 July 2016.

^b^ 2 May–10 July 2016

^c^ 14 December–3 July 2016.

^d^ The numbers are based on cases reported by a sentinel network of general practitioners and are then extrapolated [1,8].

Source: Santé publique France, French national public health agency and Regional unit Antilles Guyane, France, 2016.

Before 2016, few imported cases of ZIKV infection were reported by the National Reference Centre in mainland France, with the majority returning from French Polynesia. The number of imported cases steadily increased in 2016, reflecting the epidemic in the French departments of the Americas [1,8] (Figure 3), as observed during the chikungunya virus outbreak in 2014 [9].

## Background

Zika virus is an emerging mosquito-borne flavivirus which typically causes mild disease. Since 2015, ZIKV has spread rapidly throughout the Americas, including the French departments and collectivities [8], and revealed new ways of transmission and severe complications [10-12], including sexual transmission, congenital malformations [13,14] and neurological syndromes [15]. By 5 August 2016, 43 countries and territories had confirmed local, vector-borne transmission of ZIKV in South and Central America since 2015 [16,17].

## Discussion

Although no local mosquito-borne transmission of ZIKV has been documented in mainland France to date, criteria for local mosquito-borne transmission of ZIKV are met: a population that is immunologically naive to the virus; a high probability of introduction of the virus by travellers returning from ZIKV-affected areas; and an established competent vector. The number of returning travellers is expected to further increase over the summer months (there are approximatively 2.5 million passengers travelling by air between mainland France and Martinique, Guadeloupe and French Guiana annually [18]). In mainland France, as at 15 July 2016, 156 (25%) cases were viraemic in an area where *Ae. albopictus* is established, during the period of vector activity. These cases have the potential to trigger local vector-borne transmission in the absence of appropriate vector control measures. The findings of a study in Gabon suggest that *Ae. albopictus* played a major role in transmission of ZIKV of the African lineage [19]. However, under laboratory conditions, *Ae. albopictus* has a much lower competence for ZIKV amplification and transmission than *Ae. aegypti* (the ZIKV vector present in Americas) [20], and to date, no vector-borne transmission of ZIKV has been documented in Europe. 

The occurrence of local mosquito-borne transmission of dengue virus in 2010, 2013 and 2015 as well as chikungunya virus in 2010 and 2014 in mainland France highlights the risk of local transmission of arboviruses transmitted by *Ae. albopictus* [21-25].

The proportion of ZIKV infections that are asymptomatic is currently estimated at 80% [26]. Although the role of asymptomatic ZIKV-infected people in vector-borne transmission has not yet been formally demonstrated and quantified, a high proportion of such cases might increase the risk of local mosquito-borne transmission where *Ae. albopictus* is established and active, since most asymptomatic cases will remain undetected, and therefore no mosquito control measures will be implemented around these cases.

Eight cases of sexual transmission of ZIKV have been reported in mainland France as at 15 July 2016, including transmission by an asymptomatic man [5]. Some authors have suggested that sexual transmission may play a significant role in transmission of ZIKV and has contributed to the higher proportion of female cases observed in Brazil [27]. Case finding should therefore not only focus on travellers returning from areas with ZIKV transmission but also on their sexual partners, even in the absence of symptoms in the traveller. Cases infected by sexual transmission can initiate further vector-borne transmission, emphasising the importance of the implementation of vector control measures around all cases. The lack of knowledge on the persistence of ZIKV and the dynamics of RNA viral load in semen still pose a considerable challenge to guidance on prevention of sexual transmission of ZIKV.

Other questions remain regarding the aetiological link between ZIKV infection and neurological presentations and their spectrum [28]. Since January 2016, two cases of Guillain–Barré syndrome and one case of meningoencephalitis were reported (0.5% of all cases) in mainland France. Paraesthesia, hypoaesthesia or hyperaesthesia were reported for nine additional cases (1.5% of all cases): the frequency and relevance of these milder symptoms deserves further attention.

The expected high number of imported cases of ZIKV infection in areas where *Ae. albopictus* is established and severe ZIKV-related adverse outcomes trigger the need to monitor closely cases of ZIKV infection. Vector control measures are essential during the vector’s active period.

Furthermore, it is essential to maintain a high level of commitment of healthcare professionals, especially family practitioners, to continue their participation in surveillance and in health education. They are a major source of information for patients on the risk of ZIKV infection and for the general population on measures to prevent infection by ZIKV and other arboviruses.

## Notes

**Zika Surveillance Working Group in French departments and collectivities of the Americas:** Audrey Andrieu, Vanessa Ardillon, Lydéric Aubert, Marie Barrau, Sami Boutouaba-Combe, Luisiane Carvalho, Sylvie Cassadou, Jean-Louis Corazza, Elise Daudens-Vaysse, Frédérique Dorléans, Cécile Durand, Elise Emeville, Laurent Filleul, Noellie Gay, Guillaume Heuzé, Sylvie Lancino, Martine Ledrans, Marion Petit-Sinturel, Corinne Pioche, Véronique Servas, Lorenzo Subissi, Claudine Suivant.
